# Discrete Biochemical Systems Theory

**DOI:** 10.3389/fmolb.2022.874669

**Published:** 2022-05-04

**Authors:** Eberhard O. Voit, Daniel V. Olivença

**Affiliations:** The Wallace H. Coulter Department of Biomedical Engineering, Georgia Institute of Technology and Emory University, Atlanta, GA, United States

**Keywords:** canonical model, delay, discrete event, generalized mass action system, power-law approximation, system, stochastic event, aryl hydrocarbon receptor

## Abstract

Almost every biomedical systems analysis requires early decisions regarding the choice of the most suitable representations to be used. De facto the most prevalent choice is a system of ordinary differential equations (ODEs). This framework is very popular because it is flexible and fairly easy to use. It is also supported by an enormous array of stand-alone programs for analysis, including many distinct numerical solvers that are implemented in the main programming languages. Having selected ODEs, the modeler must then choose a mathematical format for the equations. This selection is not trivial as nearly unlimited options exist and there is seldom objective guidance. The typical choices include ad hoc representations, default models like mass-action or Lotka-Volterra equations, and generic approximations. Within the realm of approximations, linear models are typically successful for analyses of engineered systems, but they are not as appropriate for biomedical phenomena, which often display nonlinear features such as saturation, threshold effects or limit cycle oscillations, and possibly even chaos. Power-law approximations are simple but overcome these limitations. They are the key ingredient of Biochemical Systems Theory (BST), which uses ODEs exclusively containing power-law representations for all processes within a model. BST models cover a vast repertoire of nonlinear responses and, at the same time, have structural properties that are advantageous for a wide range of analyses. Nonetheless, as all ODE models, the BST approach has limitations. In particular, it is not always straightforward to account for genuine discreteness, time delays, and stochastic processes. As a new option, we therefore propose here an alternative to BST in the form of discrete Biochemical Systems Theory (dBST). dBST models have the same generality and practicality as their BST-ODE counterparts, but they are readily implemented even in situations where ODEs struggle. As a case study, we illustrate dBST applied to the dynamics of the aryl hydrocarbon receptor (AhR), a signal transduction system that simultaneously involves time delays and stochasticity.

## Introduction

Arguably the greatest challenge of systems modeling in the biomedical sciences is the choice of optimal process representations. Often the true magnitude of this challenge is ignored and the modeler either constructs an *ad hoc* model or chooses a default, such as a Lotka-Volterra system for describing the interactions among competing populations (Volterra, 1926; Lotka, 1956; May, 1973) or a mass action formulation or some variation of the Michaelis-Menten rate law for enzyme catalyzed processes (Michaelis and Menten, 1913; Voit et al., 2015). These default representations may be further extended or refined with the inclusion of environmental variables in a population model (Stein et al., 2013; Dam et al., 2020) or the inclusion of modulating effects, such as the regulation of a biochemical reaction through competitive or allosteric inhibition (Cornish-Bowden, 2012). Because there are no iron-clad rules for choosing a model, researchers often arrive at rather different formulations even for the same phenomenon. An illustrative example is the phosphofructokinase reaction in glycolysis, for which numerous rate functions of drastically different complexity have been proposed (Voit, 2017a). The choice of optimal representations becomes even more challenging at the intersections of typical biological domains, such as the combination of genetics, metabolism, and organismal physiology, because the default models of the various subdisciplines are different, thereby creating the need of multiscale models that operate at different temporal, spatial and organizational scales.

One could argue that biological processes must obey the laws of physics and that, therefore, optimal—or at least adequate—representations are prescribed. While this is true in a fundamental sense, most biological processes are so convoluted that exact physical representations of all contributing aspects become infeasible (Voit, 2008). As an example, consider the generation of two daughter cells from a bacterial mother cell. At a high level, one bacterium becomes two, two become four, and so on, and it is easy to formulate an exponential function that describes the progression well. However, if it is necessary to account for more details, for instance, in order to understand a mutant with aberrant behavior, it becomes clear that the cell division process is immensely complicated (Schafer, 1998; Carlton et al., 2020). It is multifaceted and involves so many different aspects at the molecular level that it is hardly possible to formulate the governing processes, proceeding in time and space, with elementary functions that are directly derived from the first principles of physics.

A second aspect of the challenge of biomedical systems modeling is the fact that it is usually difficult to capture the dynamics of a molecular or cellular component directly. Even the simple Michaelis-Menten rate law of enzyme kinetics does not prescribe the changing concentration of a substrate or product as the reaction progresses, but expresses the speed of the reaction as a function of the substrate concentration. By contrast, it is often feasible to characterize all influences that lead to an increase or decrease in a system component over time (Voit, 2020). Indeed, the literature contains uncounted articles about “the effect of … on …,” which explicitly or implicitly describe how a target variable changes in response to some input. Thus, this view focuses on the change in a component, rather than the state of this component, and this change is driven by the totality of all contributing factors. A natural mathematical formulation of this situation is a system of ordinary differential equations (ODEs) which, after all, equate the instantaneous change in a variable to all processes affecting this variable. Consequently, the biomathematical literature contains an enormous body of work using ODEs to analyze biological systems [for introductory texts, see (Keshet, 2005; Klipp et al., 2016; Voit, 2017b)]. Even so, it must be kept in mind that ODEs are approximations of natural processes, which are often genuinely discrete (see Supplementary Data S1, S2).

While ODEs have become the standard modeling default, the conundrum of determining the best possible model structure persists. Two generic solutions are 1) the use of *ad hoc* representations that are often chosen simply for convenience and match the natural processes sufficiently well and 2) suitable, unbiased approximations. Among the latter, linear systems are most straightforward but are often at odds with the genuine nonlinearities of biomedical systems. A prominent alternative is Biochemical Systems Theory (BST) (Savageau, 1976; Voit, 2000; Torres and Voit, 2002; Voit, 2013), which uses power-law representations for all processes, thereby creating highly structured nonlinear models in immutable, predefined formats (Supplementary Data S1).

Independent of what representations are chosen to design ODE models, the ODE format in itself faces a number of challenges. Of particular prominence among these are time delays and stochastic effects (Supplementary Data S2). Sometimes, these can be addressed with sophisticated numerical ODE solvers, but the formulation and implementation can quickly become convoluted and often requires intimate knowledge of the inner workings of these solvers.

An illustrative example for the crucial role of delays is a situation that arose when we analyzed the dynamics of anemia during malaria, a disease that is caused by *Plasmodium* parasites that invade red blood cells and eventually cause them to burst. Red blood cells furthermore disappear in large numbers due to a so-called bystander effect, in which many non-infected red blood cells perish for reasons that are not well understood. One difficult challenge that arose during our modeling attempts was the fact that red blood cells naturally have a narrowly determined life span with rather small variation; in humans, it is about 115 days ± 15% (Franco, 2012). The modeling challenge becomes apparent in the assessment of how many cells are expected to disappear at a given time point during the infection (Fonseca and Voit, 2015; Fonseca et al., 2016). Some disappear due to the infection or the bystander effect, but many are removed by the spleen because they have reached the end of their natural life. To account for the latter aspect, one needs to know the age of each cell at any given point in time. However, ODEs do not recount the ages of individual cells. Thus, the disappearance of cells from the blood stream must be based on averages, which are adequate under steady-state conditions, but not for dynamic changes caused by the growing parasite population. Even the use of delay differential equations (DDEs) is inconvenient in this case, whereas a discrete, recursive modeling approach is straightforward (Fonseca and Voit, 2015; Fonseca et al., 2016). Other pertinent examples of delays and stochasticity are presented in Supplementary Data S2 *.*

This article proposes an alternative to BST models that facilitates the modeling of genuine discreteness, delays, small numbers of components, stochastic events and combinations of these complicating factors. This alternative consists of a discrete, recursive version of BST, here dubbed “dBST,” which is straightforwardly constructed and implemented.

## Results

For the practicing computational modeler in the biosciences, a partial solution to the drawbacks of ODEs can be the use of systems of discrete-time, recursive equations, where the changes in variables are represented on the basis of power-law functions, as is the case in BST. This replacement of ODEs with recursive equations raises the immediate question whether any genuine features of ODE models are lost. The answer can be approached in two ways. First, it is rather evident that the recursive equations converge to the ODEs if the step size decreases to 0 in the limit. In fact, computer algorithms for solving ODEs use small discrete step sizes. Second, one may test whether representative nonlinear phenomena that are typically represented with ODEs, such as saturation, limit cycles, and deterministic chaos, can also be represented through recursive equations with a reasonable step size. Supplementary Data S3 discusses mathematical similarities between BST and dBST systems. Here, we focus on the response repertoire of dBST systems and their features. We also present a case study illustrating the *de novo* design of a dBST system that simultaneously accounts for both, delays and stochasticity.

### Response Repertoire of Discrete Biochemical Systems Theory Models

#### Simple Introductory Example of a Linear Pathway

The 2-variable BST system

$${\dot{X}}_{1}=2X_{0}-rX_{1}^{0.8}$$

$${\dot{X}}_{2}=\;rX_{1}^{0.8}-2.5X_{2}^{0.5}$$ (1)

**FIGURE 1:**
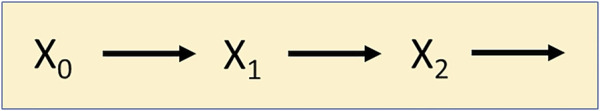
Simple linear pathway with constant input *X*_0_.

**FIGURE 2:**
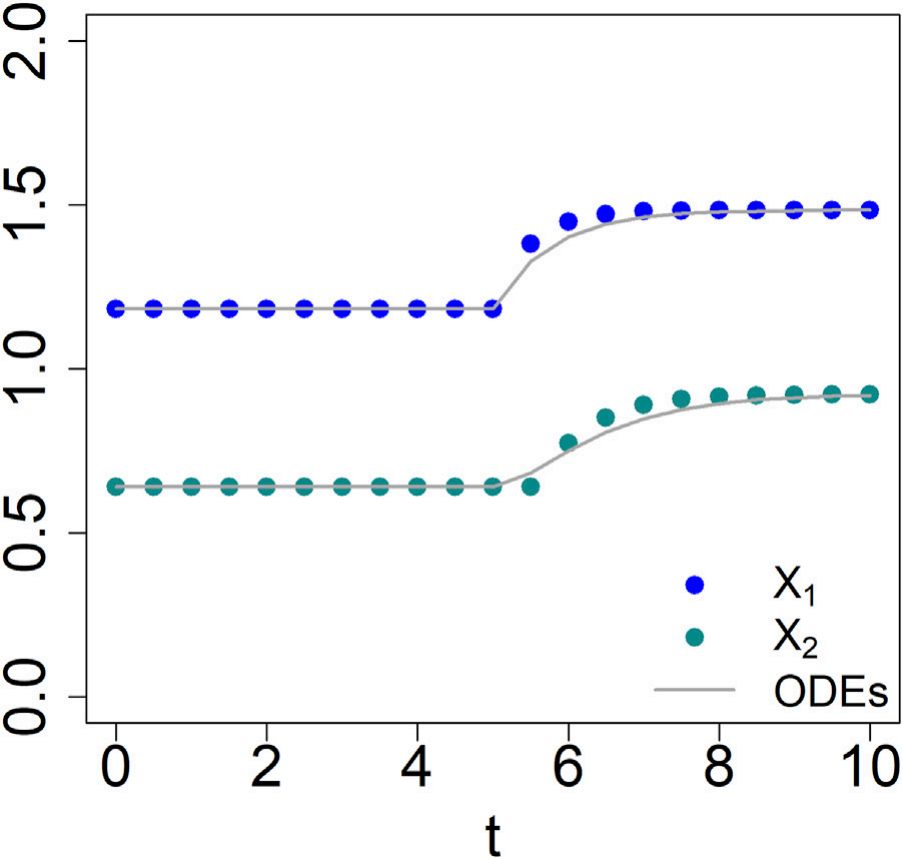
Comparison between the results of corresponding 2-variable BST (lines) and dBST (dots) models (Eqs 2, 10).

*X*_0_ = 1
*X*_1_(*t*_0_) = 1.18
*X*_2_(*t*_0_) = 0.64

represents a simple linear pathway with constant input *X*_0_ = 1 and rate *r* = 1.75 for the conversion of *X*_1_ into *X*_2_. The pathway is shown in Figure 1:

As an illustration, the system is initiated very close to its steady state (1.181653, 0.64). At t = 5, the input X_0_ is persistently increased by 20%. Solving the equations shows that the system responds to the changed input by approaching a new steady state (1.484, 0.922) (Figure 2).

The corresponding dBST system in standard notation (Supplementary Data S3) is

$$X_{1,q\cdot \vartheta }=X_{1,\left(q-1\right)\cdot \vartheta }+\vartheta \left[2X_{0}-rX_{1,\left(q-1\right)\cdot \vartheta }^{0.8}\right]$$

$$X_{2,q\cdot \vartheta }=X_{2,\left(q-1\right)\cdot \vartheta }+\;\vartheta \;\left[rX_{1,\left(q-1\right)\cdot \vartheta }^{0.8}-2.5X_{2,\left(q-1\right)\cdot \vartheta }^{0.5}\right]$$ (2a)

To simplify this notation for easier reading, we rename ${\tilde{X}}_{i}=X_{i,q\cdot \vartheta }$ and $X_{i}=X_{i,\left(q-1\right)\cdot \vartheta }$ , for *i* = 1, 2, which simplifies the appearance of Eq. 2a to

$${\tilde{X}}_{1}=X_{1}+\vartheta \;\left[2X_{0}-rX_{1}^{0.8}\right]$$

$${\tilde{X}}_{2}=X_{2}+\vartheta \left[\;rX_{1}^{0.8}-2.5X_{2}^{0.5}\right]$$ (2b)

Choosing as step size ϑ = 0.5 reveals output that is quite similar to that of the BST system, although one notes that the responses, especially of X_2_, are slightly different immediately following the switch in input (t = 5). Importantly, both formulations exhibit essentially the same dynamics and approach exactly the same steady state (Figure 2). Other step sizes yield similar results.

#### Limit Cycles

Limit cycles are representations of oscillations that are stable in a sense that, when perturbed by external influences, return to the original frequency and amplitude. Limit cycles are ubiquitous in biology (Keshet, 2005).

It has been proposed that many disease patterns can be seen mathematically as shifts from physiological to pathological limit cycles (Claude, 1995).

Like BST systems, dBST systems can capture the dynamics of stable limit cycles (Lewis and Voit, 1991; Yin and Voit, 2008). An example is the stable oscillator

$${\dot{X}}_{1}=0.011\;\left[X_{1}^{2}X_{2}^{3}-X_{1}X_{2}\right]$$

$${\dot{X}}_{2}=0.01\left[X_{1}^{-1}X_{2}^{4}-X_{1}^{3}X_{2}^{5}\right]$$ (3)

which in standard dBST format reads

$$X_{1,q\cdot \vartheta }=X_{1,\left(q-1\right)\cdot \vartheta }\;+\;\vartheta \;\cdot 0.011\;\cdot \left[X_{1,\left(q-1\right)\cdot \vartheta }^{2}\;X_{2,\left(q-1\right)\cdot \vartheta }^{3}-X_{1,\left(q-1\right)\cdot \vartheta }\;X_{2,\left(q-1\right)\cdot \vartheta }\right]$$

$$X_{2,\;q\cdot \vartheta }=X_{2,\left(q-1\right)\cdot \vartheta }\;+\;\vartheta \;\cdot 0.01\;\cdot \left[X_{1,\left(q-1\right)\cdot \vartheta }^{-1}X_{2,\left(q-1\right)\cdot \vartheta }^{4}-X_{1,\left(q-1\right)\cdot \vartheta }^{3}X_{2,\left(q-1\right)\cdot \vartheta }^{5}\right]$$ (4a)

To the human eye, this format may look rather unwieldy, but it is easily implemented into computer code. Furthermore, using the simplified notation introduced in Eq. 2, we obtain

$${\tilde{X}}_{1}=X_{1}+\vartheta \cdot 0.011\;\cdot \left[X_{1}^{2}X_{2}^{3}-X_{1}X_{2}\right]$$

$${\tilde{X}}_{2}=X_{2}+\vartheta \cdot 0.01\;\cdot \left[X_{1}^{-1}X_{2}^{4}-X_{1}^{3}X_{2}^{5}\right]$$ (4b)

**FIGURE 3:**
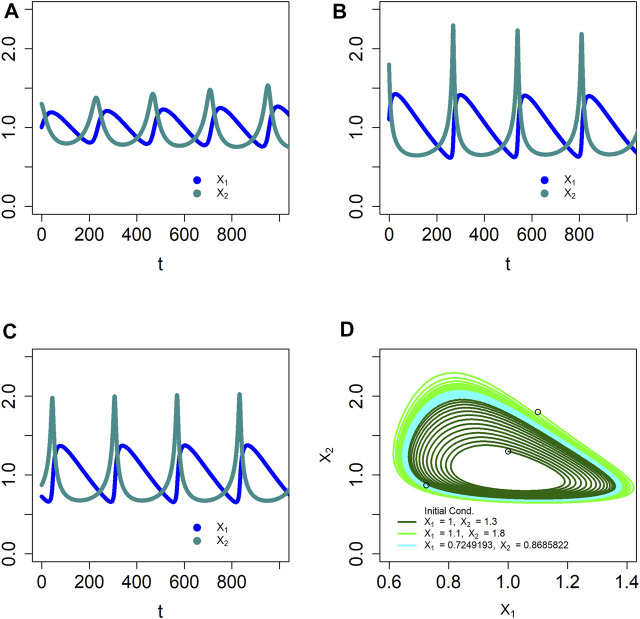
The dBST system in Eq. 4 models a stable limit cycle, as confirmed by simulations starting inside **(A)**, outside **(B)** and essentially on the limit cycle **(C)**. Panel **(D)** displays a typical phase-plane plot with superimposed oscillations spiraling out (dark green) or in (light green) toward the limit cycle, as well as starting very close to the limit cycle itself (cyan); the initial locations are indicated by circles. The trajectories appear to be smooth because the step size is rather small. The ODE model produces essentially the same solutions, even though the maximal amplitudes are slightly different.

Solving the system with step size ϑ = 0.1 confirms that the system indeed has a stable limit cycle. Namely, initial conditions inside the limit cycle, like (*X*_1,0_, *X*_2,0_) = (1, 1.3), lead to increasing oscillations, while conditions outside, such as (*X*_1,0_, *X*_2,0_) = (1.1, 1.8), generate damped oscillations; starting essentially on the limit cycle, e.g., (*X*_1,0_, *X*_2,0_) = (0.7249193, 0.8685822) demonstrates constant amplitudes. In the phase plane, the corresponding plots are outward and inward spirals, as well as the stable orbit (Figure 3). If we use the step size ϑ = 1, the system still displays a limit cycle of similar shape, but with larger amplitudes (not shown). Much larger step sizes eventually become too coarse and destroy the features of the limit cycle.

#### Deterministic Chaos

Discrete BST systems are also rich enough to permit deterministic chaos. While a rigorous proof is difficult, an example is a discrete system gleaned from the well-known Lorenz oscillator (Lorenz, 1963), which mathematician and meteorologist Edward Lorenz developed as a simplified representation of atmospheric convection, which had been modeled previously as a fluid layer for which the temperatures at the top and the bottom were kept constant at different values (Saltzman, 1962). The ODE format of this system reads:

$${\dot{X}}_{1}=0.2\cdot X_{2}-0.2\cdot X_{1}$$

$${\dot{X}}_{2}=0.6\cdot X_{1}-0.02\cdot X_{2}-0.02\cdot X_{1}\cdot X_{3}$$ (5)

$${\dot{X}}_{3}=0.02\cdot X_{1}\cdot X_{2}-0.05\cdot X_{3}$$

The reformulation into recursive equations is straightforward, and the dBST in simplified notation (see Eq. 2) reads

$${\tilde{X}}_{1}=X_{1}+\vartheta \cdot 0.2\cdot \left[X_{2}-X_{1}\right]$$

$${\tilde{X}}_{2}=X_{2}+\vartheta \cdot \left[0.6\cdot X_{1}-0.02\cdot X_{2}-0.02\cdot X_{1}\cdot X_{3}\right]$$ (6)

$${\tilde{X}}_{3}=X_{3}+\vartheta \cdot \left[0.02\cdot X_{1}\cdot X_{2}-0.05\cdot X_{3}\right]$$

**FIGURE 4:**
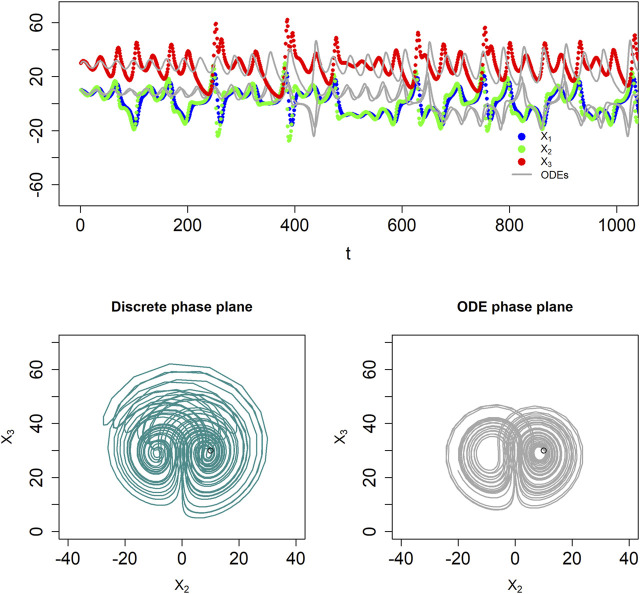
The dBST system in Eq. 6 captures deterministic chaos, similar to the ODE system proposed by Lorenz. The top panel shows results in the time domain, in comparison to the corresponding ODE system (thin grey lines). The BST and dBST systems diverge quickly (top panel), which is a genuine feature of chaotic systems. The bottom panel displays phase-plane plots of the two models, showing the discrete nature of the system in the form of connected straight lines. The initial locations are indicated with circles. Note that the maximal amplitudes of the dBST system are larger.

Solving the system with step size ϑ = 1 and initial conditions (10, 10, 30) reveals dynamics similar to that of the chaotic Lorenz equations in ODE format, both in the time domain and in phase plane (Figure 4), although the numerical details of the results are different, which is not surprising, as chaotic systems are extremely sensitive to all numerical settings, such as parameter values, initial conditions, and the step size for solving the ODEs or the discrete equations. One also notes that the maximum amplitudes of the BST system are somewhat higher than in the ODE model.

### Typical Simulations in dBST That Are More Intuitive Than in ODE Models

The simulations described in this section are straightforwardly implemented in dBST, and while it is possible to implement some of them in ODEs, such an implementation is sometimes cumbersome or difficult to intuit. Indeed, one may have to be creative if some of these issues are to be included into ODE solutions and understand the inner workings of the numerical solution algorithms. For example, the widely used Runge-Kutta method averages the slope for each solution step, and additional statements, such as if-conditions, can influence this average or cannot be taken into consideration, depending on how the solver was coded. Furthermore, using numerical solvers with variable step size requires care so that the choice of the optimal step size is not affected.

In the following, we focus on different types of stochastic events and the dependence of the system dynamics on thresholds for dependent variables. Details regarding delays are discussed in Supplementary Data S2 and in the later Case Study *Aryl Hydrocarbon Receptor Signal Transduction*.

#### Stochastic Variations in Rates

**FIGURE 5:**
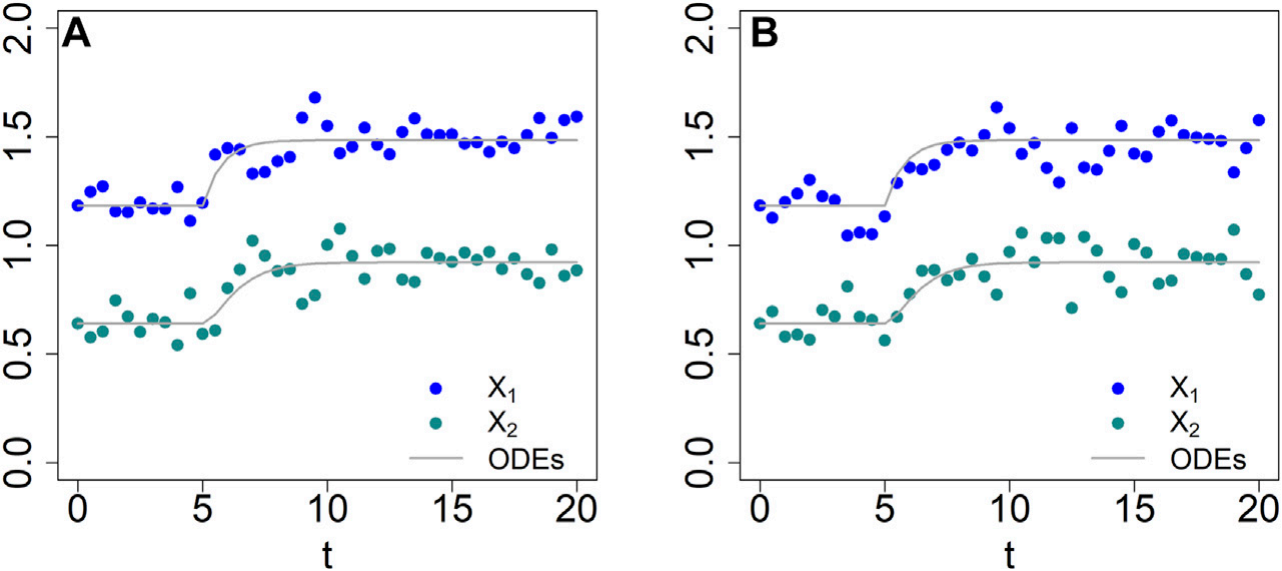
Comparison of results from the deterministic and two instances **(A and B)** of stochastic models (*ϑ* = 0.5) of the simple pathway in Figure 2. The two dBST simulations were obtained with a stochastically varying rate *r* in Eq. 1 (dots), starting from different seeds, while the solid lines are the results of Eq. 2 with constant rate *r*.

Returning to the introductory at the beginning of the *Results* section, it is easy in dBST to replace the constant rate *r* of the process converting *X*_1_ into *X*_2_ with a rate that stochastically varies within a range of, say, ± 10% of the nominal value in the example. For this illustration, we randomly sampled a rate from this range at every iteration. Two solutions are shown in Figure 5. Variations on this theme are also readily implemented. For instance, it is possible to sample a new rate less frequently than at every step.

#### Events Where System Variables Affect External Events

Suppose a signaling system responds stochastically to an environmental trigger, which is a ubiquitous situation in biological systems, especially if the concept of an “environment” includes the biophysical surroundings of cells. We develop this example in several steps, because some cases are easily addressed with ODEs, whereas others are not. In the simplest case of a stochastic environmental input it is possible to include if-statements into a numerical solver, such as the deSolve R package (Soetaert et al., 2010), which was designed to solve various initial-value problems, differential algebraic equations and partial differential equations. However, if one or more of the system variables influence the random variable, the situation is much more complicated, as the random variable must be adressed inside the solver, which is difficult for a ODE solver but straightforward in the discrete case.

**FIGURE 6:**
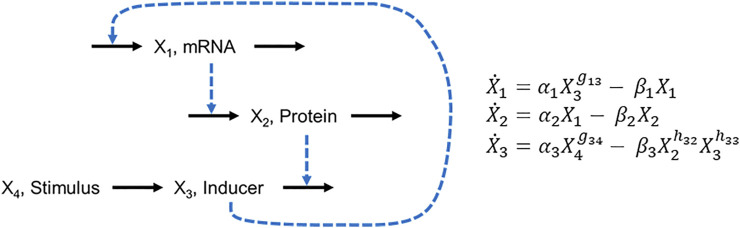
Diagram of the lac operon and corresponding equations, as proposed by Savageau (2001).

Suppose at first that the environmental trigger is present or absent for stochastically long time periods that begin at random time points and whose magnitude affects the response of the signaling system. As a specific example, consider the lac operon of the bacterium *E. coli*, where external lactose triggers changes in gene expression (Lewis, 2005). Savageau (2001) proposed a model of the system in the form of the diagram in Figure 6 and represented it with S-system equations. In this model, *X*_1_ is the concentration of mRNA of the lac operon, *X*_2_ is the concentration of the enzyme β-galactosidase, which catalyzes the conversion of lactose into galactose and glucose, and *X*_3_ and *X*_4_ are the intracellular and extracellular concentrations of lactose, respectively. *X*_4_ is considered an independent variable and therefore that does not require its own differential equation.

Savageau discussed the kinetic orders (g and h parameters) but did not provide specific parameter values for them or for the rate constants (α and β parameters). We use this example for a series of demonstrations, specifying the parameter values as shown in Eq. 7.

$${\dot{X}}_{1}=0.2X_{3}^{2}-0.1X_{1}$$

$${\dot{X}}_{2}=0.5X_{1}-X_{2}$$ (7)

$${\dot{X}}_{3}=0.1X_{4}-0.1X_{2}X_{3}$$

**FIGURE 7:**
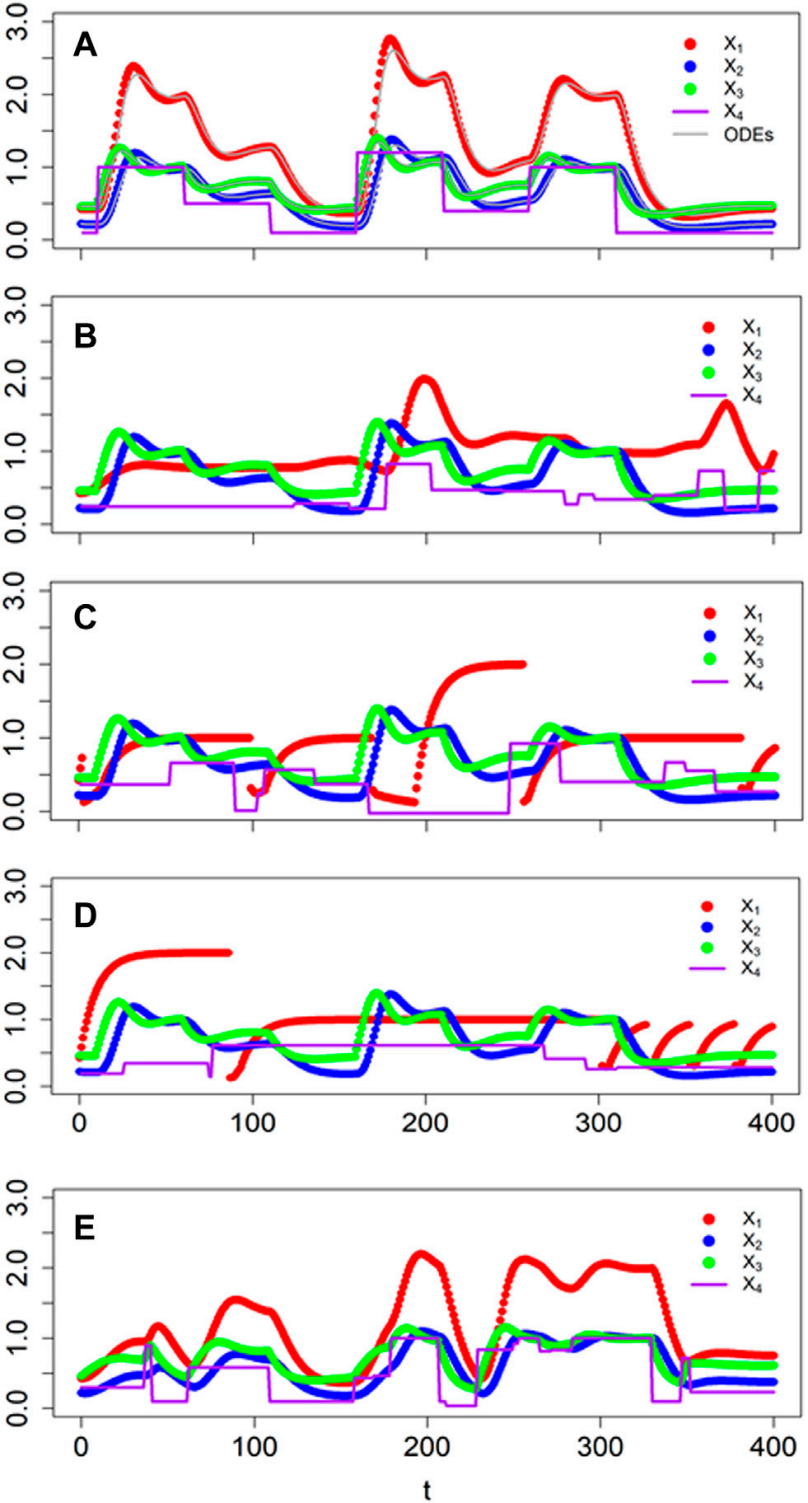
Comparison of responses of BST (faint grey lines) and dBST models (dots) to perturbations in external lactose concentraion (solid lavendar line). **(A)** Timing and magnitudes are known *a priori* (Table 1). **(B)** Signaling events occur in a stochastic manner and signal magnitudes are random within a given range (see Text for details). **(C,D)** Two sets of responses, where switches in the equation of *X*_1_ depend not only on the stochastic input *X*_4_, but also on the value of *X*_3_. **(E)** The value of *X*_3_ is used to generate a success probability for a Bernoulli random variable. Specifically, if the Bernoulli process returns 1, the new value for *X*_4_ is given by a truncated normal with mean 0.5 and a standard deviation equal to the value of *X*_1_.

For the first demonstration, suppose that the stimulus, external lactose (*X*_4_), is available in irregular time periods and concentrations that vary randomly within reasonable ranges. If the switch points and magnitudes of *X*_4_ are known beforehand, the simulation of the ODE system is straightforward. For instance, if the system starts at its steady state (0.43, 0.22, 0.46) with *X*_4_ = 0.1 and the stimulus changes according to Table 1, one directly obtains the output in Figure 7A.

**TABLE 1:** A priori known schedule of switches in stimulus in the lac operon model.

Time	0	10	60	110	160	210	260	310
Stimulus	0.1	1	0.5	0.1	1.2	0.4	1	0.1

If the timing and magnitudes are stochastic, a simulation with a standard ODE solver can be cumbersome as one needs to know how to evaluate functions of time inside the algorithm. Nonetheless, this situation can still be addressed, for instance, with deSolve or in Matlab. This case is again straightforwardly implemented in a dBST system. One set of results is shown in Figure 7B for event times sampled from an exponential distribution with a rate of 1/60. The magnitude of the signal at every event was sampled from a normal distribution with mean μ = 0.5 and standard deviation σ = 0.25.

As the next phase of the example, we analyze the situation discussed by in Savageau (2001), where the format of the first ODE in Figure 6 depends on the current value of *X*_3_. Specifically, the author defined

$${\dot{X}}_{1}=\{\begin{array}{cc} \alpha_{1L}-\beta_{1}X_{1} & if\;X_{3}<X_{3L}\\ \alpha_{1}X_{3}^{g_{13}}-\beta_{1}X_{1} & if\;X_{3L}\leq X_{3}\leq X_{3H}\\ \alpha_{1H}-\beta_{1}X_{1} & if\;X_{3}>X_{3H} \end{array}$$ (8)

where *X*_3*L*_ and *X*_3*H*_ are threshold values and the corresponding “low” and “high” rate constants *α*_3*L*_ and *α*_3*H*_ are different. While it is possible to address this task by embedding if-conditions into an ODE solver, these situations of thresholds are much more easily called up in dBST: The If-statements are directly implemented in the recursive step for variable *X*_1_. As a demonstration, suppose again that the external trigger changes in unpredictable patterns, which we assume to be random in terms of timing and magnitude, as before, and that the thresholds are in effect. Two typical results are shown in Figures 7C,D.

As the most complicated variation of the example, let us now suppose that both the magnitude and frequency of the stochastic events depend on the state of the system. For instance, the amount of external lactose *X*_4_ to be imported into the cell could stochastically depend on the current mRNA prevalence *X*_1_ and also the internal lactose concentration *X*_3_, which together could have an effect on the characteristics of the import transporters. This situation cannot easily be addressed with an ODE solver, if at all, as one can no longer first calculate the current level of *X*_4_ and then present it to the solver as a time-dependent function. Instead, such a situation mandates that the stochastic variables be evaluated inside the solver, a process that can interfere with the ODE solution. By contrast, the discrete version is easily implemented. An example is presented in Figure 7E.

### Parameter Estimation

Much has been written about the estimation of parameter values of ODE systems from time series data [*e.g.,* see reviews (Gennemark and Wedelin, 2007; Chou and Voit, 2009; Gennemark and Wedelin, 2009; Gábor and Banga, 2015)]. In order to make typical gradient methods and evolutionary algorithms more efficient, it has been suggested to smooth the time series data, use points from the smoothed trends, and estimate slopes of the time trends corresponding to the chosen points (Varah, 1982; Voit and Savageau, 1982; Voit and Almeida, 2004). Substituting these quantities in the ODE system converts the task of estimating parameter values for ODEs into an estimation from algebraic equations. Thus, for estimation purposes, the ODE for each variable *X*_*i*_,

$${\dot{X}}_{i}=F_{i}\left(X_{1},\;\ldots ,\;X_{n}\right)\;\;\mathrm{i = 1, ...,}n$$ (9)

is converted into a system of *K* algebraic equations of the format

$$S_{i}\left(t_{1}\right)=F_{i}\left(X_{1}\left(t_{1}\right),\;\ldots ,X_{n}\left(t_{1}\right)\right)$$

$$S_{i}\left(t_{2}\right)=F_{i}\left(X_{1}\left(t_{2}\right),\ldots ,X_{n}\left(t_{2}\right)\right)$$ (10)

$$S_{i}\left(t_{K}\right)=F_{i}\left(X_{1}\left(t_{K}\right),\;\ldots ,X_{n}\left(t_{K}\right)\right)$$

Here, each equation corresponds to one chosen time point. The *X* values are either the raw data or the corresponding data from the smoothed time trend, while *S* indicates the corresponding slope of the time course.

This method of using estimated values and slopes tends to be computationally much more efficient than parameter inferences directly from ODEs, for instance with a gradient method or an evolutionary algorithm (Voit and Almeida, 2004). One drawback is that the estimation of slopes exacerbates noise in the data (Knowles and Renka, 2014; Voit, 2017b). To some degree, this problem is alleviated by smoothing the data appropriately.

For the discrete system, no slopes need to be estimated as the difference to be used instead, $X_{i,q\cdot \vartheta }-X_{i,\left(q-1\right)\cdot \vartheta }$ , is directly obtained from the data. Thus, given measurements for all *X*_*i*_ at different time points, and possibly a smoothing step, the estimation of the parameters of a dBST system is straightforward.

**FIGURE 8:**
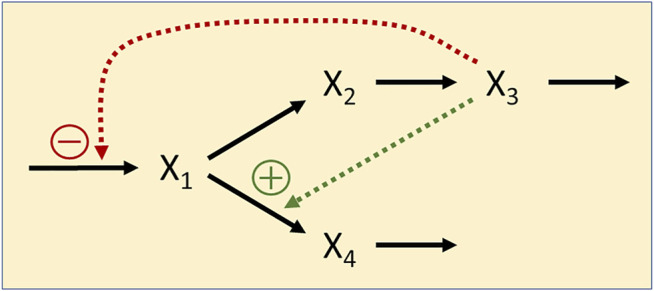
Simple branched feedback with dual regulation by *X*_3_.

As an example, consider the branched pathway in Figure 8, for which we pretend to have experimental measurements that had been smoothed, for instance, with a spline. For the illustration, we actually created synthetic “data” from a GMA (BST) model in ODE format and did not worry about noise, in order to assess most clearly to what degree BST and dBST models correspond and reflect the synthetic data. Analogously to the BST model, the format of the dBST equations is dictated directly by the flow structure and regulation of the pathway. In the simplified notation of Eq. 2b, the dBST equations take the form

$${\tilde{X}}_{1}=X_{1}+\vartheta \cdot \left[a_{1}\cdot X_{3}^{g_{1}}-b_{1}\cdot X_{1}^{h_{11}}-c_{1}\cdot X_{1}^{h_{12}}\cdot X_{3}^{h_{13}}\right]$$

$${\tilde{X}}_{2}=X_{2}+\vartheta \cdot \left[b_{1}\cdot X_{1}^{h_{11}}-b_{2}\cdot X_{2}^{h_{2}}\right]$$

$${\tilde{X}}_{3}=X_{3}+\vartheta \cdot \left[b_{2}\cdot X_{2}^{h_{2}}-b_{3}\cdot X_{3}^{h_{3}}\right]$$ (11)

$${\tilde{X}}_{4}=X_{4}+\vartheta \cdot \left[c_{1}\cdot X_{1}^{h_{12}}\cdot X_{3}^{h_{13}}-c_{2}\cdot X_{4}^{h_{4}}\right]$$

and we suppose that the values of the parameters are unknown. We analyze datasets with different densities of observation time points. For each parameter optimization, we use the optim function in R (R Core Team, 2018), which is based on the Nelder-Mead method (Nelder and Mead, 1965). Multiple sequential optimizations were performed for each example and the process was stopped when the difference of consecutive errors was less than 10^–3^.

**FIGURE 9:**
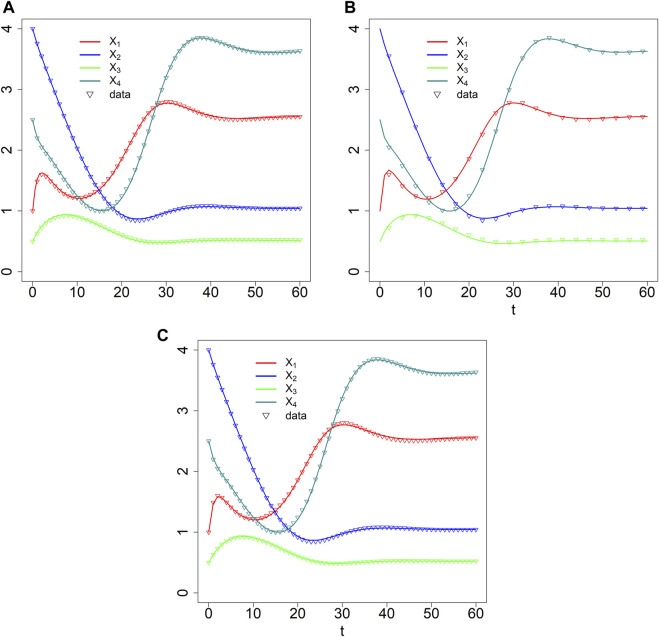
Data fits with dBST [*τ* = 1 **(A)** and *τ* = 3 **(B)**] and BST **(C)** models.

For the first illustration, we suppose that data had been obtained in intervals of *τ* =1, that is, for *t* = 0, 1, 2, ..., 60, and define ϑ = 1. The estimation result, shown in Figure 9A, captures the data well. The associated residual error, divided by the total number of data in the four time courses (4 *n*) is SSE/4 *n* = 0.1007525/(4 * 61) = 4.13 × 10^–4^. Fitting the same data, but with step size ϑ = 0.5 (results not shown), the fitting error is roughly halved, with SSE/4 *n* = 0.0443176/(4 * 61) = 1.81 × 10^–4^.

As a second illustration, we assume that the data are much sparser (*τ* =3), that is, with measurements obtained at time points *t* = 0, 3, 6, 9, … , 60; we again define ϑ = 1. The result is shown in Figure 9B. The model still fits the data well, with a residual error, divided by the total number 4 *n* of data, of SSE/4 *n* = 0.03697914/(4*21) = 4.40 × 10^–4^, which is slightly, but not substantially higher than for the denser dataset. The estimated parameter values are slightly different from those obtained for the denser dataset, which is not surprising. However, it is interesting that the sparsity of the data hardly seems to affect the estimation.

Quasi as a baseline for comparison, we also fit the synthetic data with ODE equations in GMA format. They also recapture the data well (Figure 9C), even though the estimated parameter values are not identical to those used to create the data (Table 2), indicating some numerical redundancy among the parameters. The residual error, divided by the number of data is SSE/4 *n* = 0.03966285/(4*61) = 1.62 × 10^–4^, which is again in the same range as for the discrete model. The parameter values are slightly different from those estimated with the dBST model. This result is to be expected because the meaning of each multiplicative parameter is, strictly speaking, not identical for BST and dBST models, as the former represent instantaneous rates and the latter stepwise changes.

**TABLE 2:** Parameter estimates obtained for different settings of the dBST model in Eq. 11 and the corresponding BST model.

Parameter	Value for “data” generation	dBST (*τ* =1) ϑ = 1	dBST (*τ* =1) ϑ = 0.5	dBST (*τ* = 3) ϑ = 1	BST
X_0_	1	0.981337	0.826518	0.951492	1.553729
a_1_	0.25	0.263397	0.279577	0.263219	0.15743
b_1_	0.08	0.166199	0.132337	0.159418	0.140825
b_2_	0.2	0.276071	0.245437	0.265376	0.249848
b_3_	0.3	0.395566	0.355906	0.386093	0.351592
c_1_	0.15	0.091586	0.091028	0.089742	0.097641
c_2_	0.25	0.169922	0.172505	0.166313	0.173117
d_1_	0.1	4.775786	10.00496	8.298336	−0.02349
g_1_	−2	−1.71411	−1.94822	−1.76486	−2.03513
h_11_	1	0.55773	0.677036	0.56065	0.625422
h_12_	2	1.736943	2.072465	1.794026	2.459446
h_13_	0.5	−0.29347	0.072883	−0.24301	0.619297
h_2_	0.4	0.327098	0.355548	0.345685	0.358772
h_3_	0.6	0.498803	0.531523	0.525627	0.504745
h_4_	0.8	0.935483	0.968566	0.950606	1.038234
SSQ/n		4.13 × 10^–4^	1.81 × 10^–4^	4.40 × 10^–4^	1.62 × 10^–4^

### Case Study: Aryl-Hydrocarbon Receptor Signal Transduction

The aryl-hydrocarbon receptor (AhR) is a highly conserved sensor for specific cues during development and normal physiology (Stockinger et al., 2014; Brinkmann et al., 2019; Zhu et al., 2019), as well as for external, xenobiotic compounds (Stevens et al., 2009; Simon et al., 2015) or danger signals derived from the invasion of parasites, which are mediated through compounds like the tryptophan-derivative kynurenine (Julliard et al., 2014; Gupta et al., 2022). In response to such signals, the AhR signal transduction system triggers the upregulation of a host of genes, most prominently those coding for cytochrome P450 enzymes that metabolize toxicants.

**FIGURE 10:**
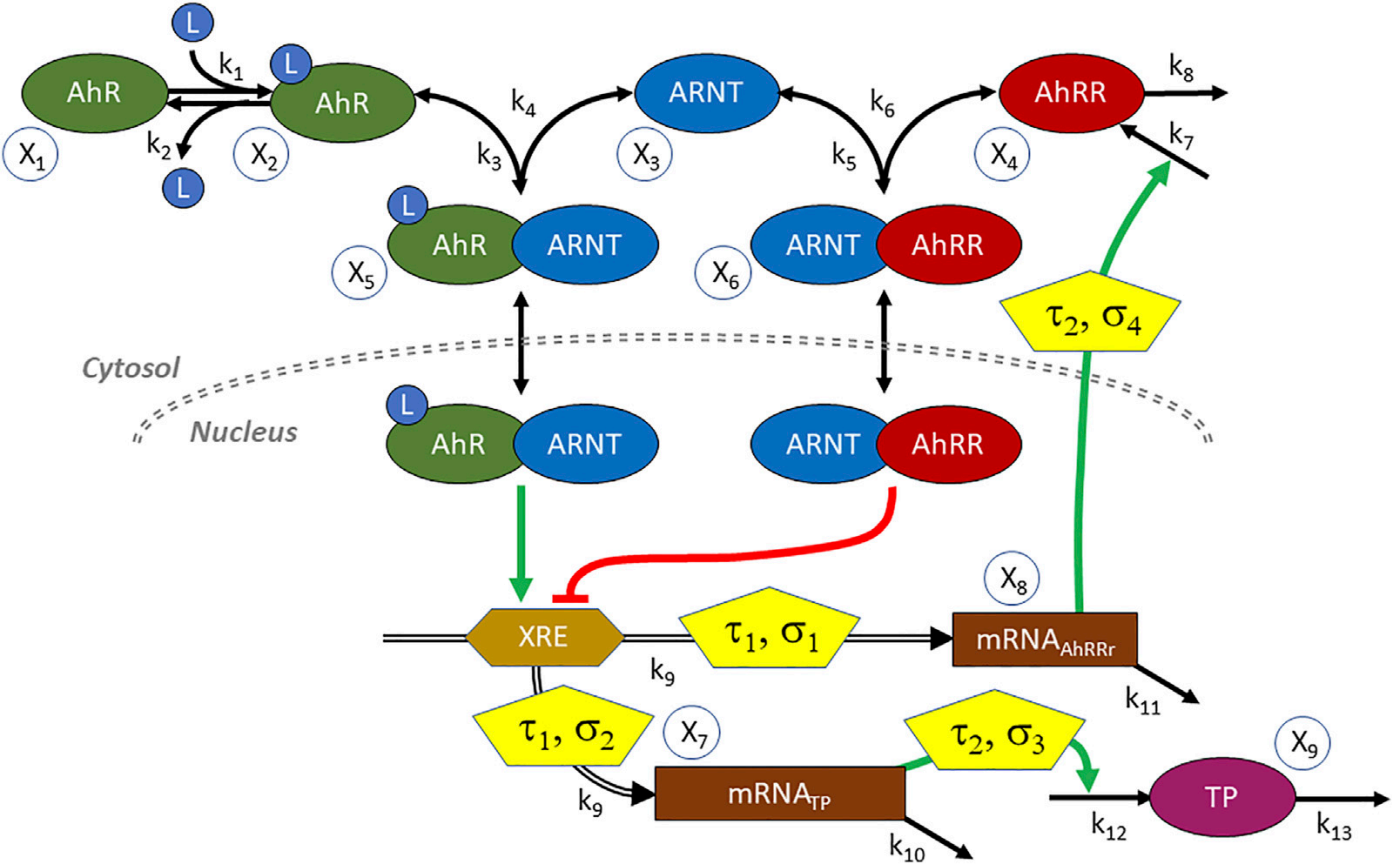
Diagram of the AhR signal transduction system. AhR is activated by a ligand L and binds to the nuclear translocator ARNT. The complex serves as a transcription factor of genes whose promoter regions contain the xenobiotic response element XRE. These genes code for a variety of target proteins (TP) including, notably, the AhR repressor AhRR. AhRR competes with AhR for ARNT, and the complex inhibits gene expression. Transcription and translation incur delays (*τ*_1_, *τ*_2_) and are stochastic in nature (*σ*_1_, ..., *σ*_4_). L is also considered to be stochastic.

The generic functionality of the AhR-system is depicted in Figure 10. Once a ligand (L) binds to AhR, the activated AhR forms a complex with the AhR nuclear translocator ARNT. This complex translocates to the nucleus, where it serves as a transcription factor that binds to the xenobiotic response element XRE—or a non-canonical XRE analog—within the promotor regions of numerous inducible target genes (Huang and Elferink, 2012). The AhR repressor AhRR competes with AhR for ARNT (Evans et al., 2008). Intriguingly, the gene coding for AhRR is itself under the control of the AhR-ARNT transcription factor, thereby creating a negative feedback loop that eventually stops the expression of AhR-ARNT controlled genes (Zudaire et al., 2008). As one might expect, reality is more complicated, for instance, due to compounds like the hypoxia inducible factor-1α (HIF1α) that compete with AhR and AhRR for ARNT (Spence et al., 1970) and to several cofactors modulating the process (Simon et al., 2015), but the AhR-ARNT-AhRR system by itself contains enough interesting complexity for the present illustration.

One issue in setting up an ODE model is the substantial time delay between transcription factor binding, the actual availability of AhRR, and the resulting repression of target gene expression (Koussounadis et al., 2015). In yeast and mouse, this type of delay was found to be at the order of 3–6 h (Fournier et al., 2010) (Liu et al., 2016). A delay of this magnitude is crucial in the AhR system, as it noticeably delays the inhibitory effect of AHRR on target gene expression.

A second issue is the fact that transcription and translation are known to be stochastic processes (Raj and van Oudenaarden, 2008). In fact, at least in some cases, activation of a promotor causes the production of proteins to occur in short bursts and yields variable protein amounts that occur at random time intervals (McAdams and Arkin, 1997). Delays and stochasticity of course are not mutually exclusive but occur at the same time (Gedeon and Bokes, 2012). A model of this stochasticity for the case of AhR, using a discrete model based on the Gillespie algorithm, was presented by Simon et al. (2015). However, it did not explicitly account for the time delays between AhR binding and target protein expression and the role of AhRR as repressor.

Taken delays and stochasticity into account, we can formulate a dBST model that allows us to test the effects of delay and stochasticity. In mass-action and power-law format, and with simplified notation (see Eq. 2b),such a model has the following format: 
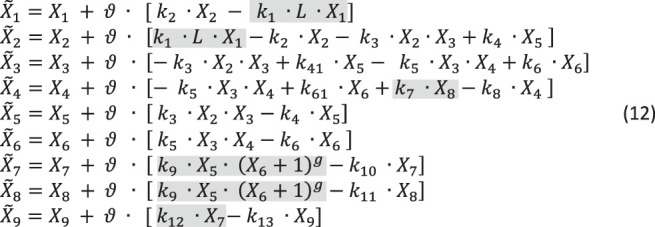


The variable names are defined in Figure 10. For this illustration, we choose reasonable rate constants as shown in Table 3 and set the inhibition parameter as *g* = −4; to avoid numerical issues for *X*_6_ = 0, we define the inhibition as ${\left(X_{6}+1\right)}^{g}$ . The simulations start at the steady state in the absence of ligand (*L* = 0), which is (*X*_10_, ..., *X*_90_) = (10, 0, 10, 0, 0, 0, 0, 0, 0). The shaded terms are subject to delay, stochasticity, or both (see Figure 10). Time is roughly in hours. The step size was taken as ϑ = 0.1.

**TABLE 3:** Parameter values for the AhR signal transduction system.

*k*_1_	*k*_2_	*k*_3_	*k*_4_	*k*_5_	*k*_6_	*k*_7_	*k*_8_	*k*_9_	*k*_10_	*k*_11_	*k*_12_	*k*_13_	*g*	*τ*_1_	*τ*_2_
1	2	1	1	1	2	2	2	10	10	10	10	6	−4	3	4

**FIGURE 11:**
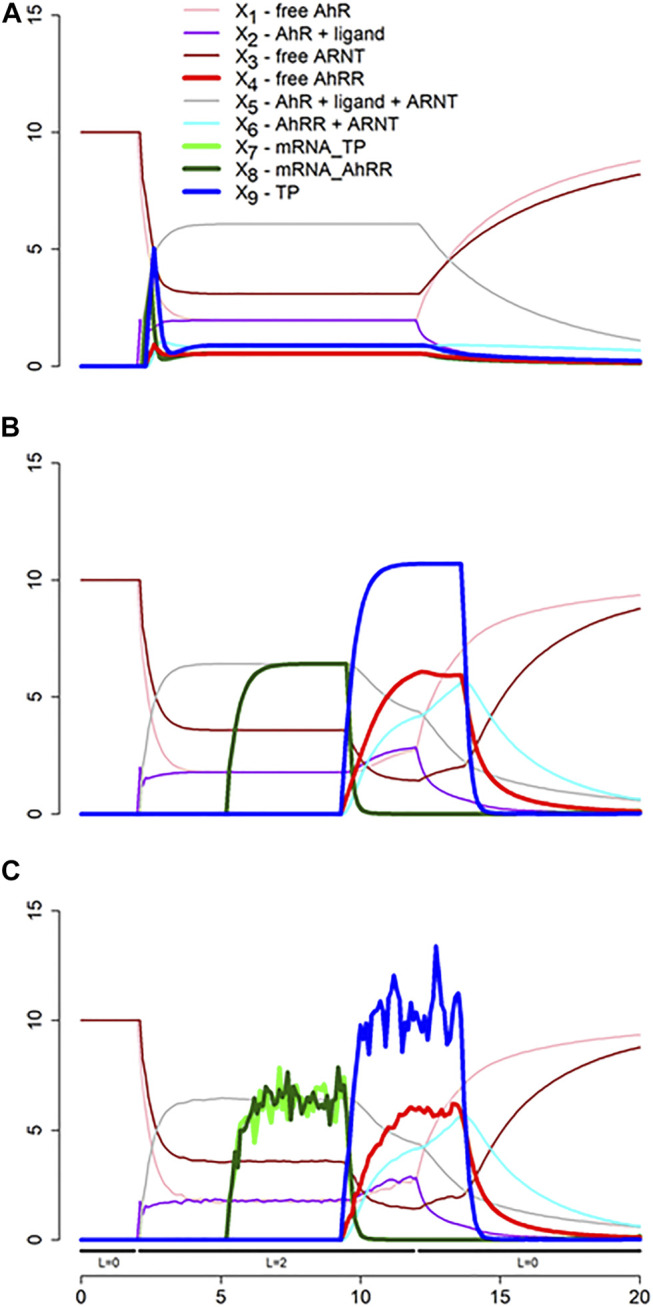
Simulation results of three scenarios. **(A)** Time delays and stochasticity are simply ignored. **(B)** Delays for transcription and for translation and activation of protein are taken into account. Note that the dynamics of *X*_7_ and *X*_8_ is the same. **(C)** Ligand availability, transcription and translation are considered stochastic. See Text for further details.

We show the result of three scenarios. In the first, the time delays and any stochasticity are simply ignored (Figure 11A). The second scenario accounts for two types of delays (Figure 11B), one for transcription and one for translation and activation of protein, and the third incorporates both, delays and noise (Figure 11C). For simplicity, we assume the same delay for the transcription (*τ*_1_ = 3 h) and translation and activation (*τ*_2_ = 4 h) of AhRR and a representative target protein (TP), even though these delays are in reality protein-specific (Koussounadis et al., 2015). Also for simplicity, we assume the same stochastic structure for *σ*_1_ and *σ*_2_ and for *σ*_3_ and *σ*_4_ (see Figure 10). Specifically, these stochastic events are modeled with values from the normal distribution *N* (1, 0.1), which are multiplied to the affected fluxes. We also added stochasticity to the ligand availability; it did not have much effect but shows up in the dynamics of *X*_1_, ..., *X*_4_.

Both simulations start at the steady state without ligand (L = 0). At time t = 2, the ligand concentration is set to 2, and at t = 12 it is returned to 0. The result of the first scenario simulation (Figure 11A) reveals that the production of target protein (TP) very briefly peaks, but that not much TP is produced, due to the immediate onset of inhibition by AhRR. By constrast, accounting for time delays yields a dramatically different dynamics (Figure 11B): Critically, the time delays permit transcription and translation to occur unabatedly until the repression sets in. Specifically, after 3 h, mRNA becomes available, and after an additional 4 h, proteins emerge, including AhRR, which quickly binds to ARNT and begins repressing transcription, resulting soon after in decreased protein production. If the ligand is available beyond time t = 12, the production of protein oscillates, and as soon as the ligand is no longer present, the system returns to the original steady state (not shown). The results for an ODE model without delays and stochasticity are essentially the same as in Figure 11A, and a delay differential equation model, ignoring stochasticity, produces more or less the same results as in Figure 11B. The combination of delays and stochasticity is difficult to capture with differential equations, but it is easily implemented in dBST Figure 11C.

## Discussion

Modeling approaches utilizing the framework of Biochemical Systems Theory (BST) have proven powerful in biomedical systems analysis for over seven decades (Savageau, 1969a; Savageau, 1969b; Savageau, 1970). Our goal in the present article was a demonstration that discrete BST (“dBST”) models are noteworthy alternatives to ODE-based BST systems and that they can shed light on complex biomedical phenomena in a similar manner. Discrete dBST models are arguably also more intuitive to newcomers coming from the field of biology, for whom differential equations are often an obscure and dreaded domain of insider mathematics.

The proposal of using dBST is most certainly not a call for abandoning systems of ODEs in biomedical models. ODEs have proven immensely beneficial in all of science, and biomedical applications are no exception. Nonetheless, there are situations that are difficult to align with the concept of instantaneous change. Examples include genuinely discrete events, delays, and stochastic phenomena affecting the phenomenon under study. For instance, we showed elsewhere, in the context of red blood cell death during malarial anemia, that the precise dynamics of blood infections is very difficult to capture with ODEs, but straightforward to implement in a discrete-recursive model (Fonseca and Voit, 2015; Fonseca et al., 2016). Similarly, we demonstrate here and in the Supplementary Data S2 that delays and internal or external stochastic influences affecting a dynamical system are often more easily incorporated into discrete rather than differential equations.

Many of the advantages of BST as a tool for model selection and analysis translate directly into its discrete analog, dBST. Whereas it is generally difficult to choose the most appropriate mathematical formats for representing ill-characterized phenomena *a priori*, BST and dBST offer guidance at the very beginning of the modeling process, where it is most urgently needed. At the very least, the use of power-law functions offers a viable, unbiased starting point. The power-law format used in BST and dBST is no panacea, but it is a local approximation of mathematically guaranteed quality that typically has a wider range of validity than linear formulations and, embedded into ODEs, is provenly rich enough to permit the inclusion of any differentiable nonlinearities (Voit and Savageau, 1986; Savageau and Voit, 1987).

The use of dBST instead of BST does not create practical design or implementation problems *per se*, and paradigmatic nonlinearities, such as limit cycles and chaos, can be captured in dBST, as we demonstrated here. If the goal of a dBST model is to mimic a corresponding ODE system as closely as possible, a small step size may have to be chosen. For instance, in the example of limit cycles, a larger step size retained the basic structure and shape of the limit cycle system, but the numerical features were clearly affected. However, the typical task in practical applications is not to create an analog of an ODE system but to convert observed data, together with contextual information, into a computable structure. This inference process is actually simpler in dBST than BST, as most biomedical phenomena are naturally discrete and the determination of optimal parameter values does not require the estimation of slopes.

We demonstrated the ease of designing a dBST model with several small examples and with a moderately complex signal transduction system that triggers changes in gene expression following an exposure to specific toxicants or internal ligands. This phenomenon is difficult to capture with an ODE model because it is critically affected by substantial time delays, which are comingled with the well-known stochastic nature of gene transcription and translation. Our analysis makes it evident that these aspects must not be ignored lest erroneous results are obtained. It also shows how straightforward it is to incorporate these aspects into a dBST model.

## Data Availability

Publicly available datasets were analyzed in this study. This data can be found here: https://github.com/LBSA-VoitLab/dBST.
