# Supplementary material for: Discrete Biochemical Systems Theory
Source: Front Mol Biosci. 2022 May 4;9:874669. doi: 10.3389/fmolb.2022.874669 (PMC9116487; doi:10.3389/fmolb.2022.874669)
Supplement: Supplementary file 1 [file DataSheet1.PDF]

Supplements

Discrete Biochemical Systems Theory (dBST)

Eberhard O. Voit and Daniel V. Olivença

S1. Choosing the Right Model

Essentially every model analysis of biomedical data begins with the selection of an appropriate mathematical representation. Whether an ODE model or a recursive formulation is chosen, the appropriate choice of functions for the right-hand sides of the equations is a difficult challenge. This challenge is often ignored by using functions that seem reasonable, but it is in truth daunting and unsolved, as there is seldom unbiased guidance toward the most appropriate choices. A powerful alternative to physics-based mechanistic descriptions or default assumptions is a suitable approximation. The simplest option is linearization, which is very convenient from a mathematical and computational point of view and has been enormously successful for analyses of engineered systems. By contrast, linear models are often limited in their ability to capture the essence of natural biomedical phenomena, which are always saturated and often display nonlinear dynamics, such as threshold behaviors, stable oscillations, and even chaos [1].

A natural extension of a linearization appears to be a second-order Taylor series. However, while this nonlinear option is simple for a single variable, it becomes practically intractable for systems of moderately realistic size [2]. By contrast, the power-law approximation is an excellent compromise between theoretical rigor and practicality, even for large systems. It is the fundamental formulation of processes in the modeling framework of Biochemical Systems Theory (BST; for books and reviews, see [3; 4; 5; 6; 7]).

To formulate a BST model with *n* dependent variables X_i, *n* ODEs are set up, each containing T_i processes of synthesis or degradation, and all these processes are formulated individually as power-law terms. The result is a so-called Generalized Mass Action (GMA) system of the form

$$\dot{X}_i = \sum_{k=1}^{T_i} \pm\gamma_{ik} \prod_{j=1}^{n} X_j^{f_{ikj}} \qquad i = 1,2,\ldots,n. \qquad \text{(S1)}$$

Here, the γ-parameters are rate constants and the *f*-parameters are kinetic orders that characterize the strength of the effect of a variable on the process in which it is involved. It is possible to include independent variables in the same format, but these do not have their own ODEs [6].

A variant of this GMA formulation is the S-system format, in which all processes augmenting or producing a variable are first aggregated into a single sum per equation and then collectively

modeled with one power-law term. Analogously, all processes diminishing or consuming the variable are aggregated and then formulated as a power-law term. As a result, every S-system consists entirely of ODEs with at most one balance between two power-law terms on the right-hand side of each equation, each typically containing several variables [8]. For *n* dependent variables, the typical S-system format is

$$\dot{X_i} = \alpha_i \prod_{j=1}^{n} X_j^{g_{ij}} - \beta_i \prod_{j=1}^{n} X_j^{h_{ij}} \quad i = 1,2,\ldots,n. \qquad \text{(S2)}$$

Similar to GMA systems, the α's and β's are rate constants and the *g*'s and *h*'s are kinetic orders.

The power-law representation constitutes an intriguing compromise. On the one hand, it is nonlinear and able to capture a much wider range of dynamic responses than a linear model (*e.g.*, [9; 10]). This property of nonlinear flexibility is well suited and indeed necessary for the description of biomedical processes. On the other hand, the power-law representation is linear in logarithmic coordinates and, along with the linear structure of sums and differences of terms, simplifies many analyses of these systems (*e.g.*, [8; 11; 12]). Importantly, this feature is true for any number of variables. Of particular note is that the S-system format permits the computation of steady states with purely algebraic means [6; 8], which is very rare among nonlinear models outside Lotka-Volterra systems [13; 14; 15].

It is noted that the GMA format is a direct generalization of the well-known mass-action representation [16; 17; 18; 19]. However, it permits the easy inclusion of non-integer kinetics and regulatory processes, which are not explicitly accounted for in the original mass-action format.

Savageau, who first proposed BST [8; 20; 21] and further developed this modeling framework over five decades, offered the following rationale for the choice of the power-law format:

> "The ultimate purpose in developing this formalism is to provide an explanation for the behavior of large-scale biochemical systems rather than individual reactions… However, as yet, no method of systems analysis has been proposed which takes into account the particular non-linear nature of biochemical systems… For example, it is well-known that a linear model will not exhibit sustained oscillations that are stable with respect to changes in amplitude… [Furthermore,] the available methods of kinetic analysis are inadequate for obtaining the complete rate law of complex regulatory enzymes, and even if these methods were available, the amount of experimental data for such an analysis might be a more basic limitation. The approximation philosophy here is very similar to that used in the linear modeling of many physical systems. The non-linear approximation, however, has two distinct advantages. First, the range of concentration over which the approximation is valid is considerably greater than that in the linear case. Second, the non-linear approximation is capable of exhibiting many of the more interesting responses typical of biological systems, and therefore, it represents a better model of these phenomena than a linear approximation…"

The power-law approximation is a local approximation with simple curvature that is mathematically guaranteed to represent the approximated function very well within the vicinity of a chosen operating point, but may deviate considerably farther away from this point. These

facts, together with the rigid format of power-laws, might suggest that the repertoire of nonlinear responses that these functions can represent is rather limited. However, this conclusion is faulty if power-laws are used within ODEs, and it has been shown that in fact any differentiable nonlinearity can be captured exactly with BST models, both in S-system and GMA format [9; 10]. Such nonlinearities include saturation and threshold phenomena, as well as stable limit-cycle oscillation and deterministic chaos.

As a consequence of the wide repertoire of nonlinear responses, these models have moved far beyond biochemical systems and found application in various branches of biology and medicine (*e.g.*, [22; 23; 24; 25]), as well as in a variety of other fields of science, mathematics, statistics, and beyond (*e.g.*, [26; 27; 28; 29; 30; 31; 32; 33; 34; 35]). A comprehensive review has described some of these applications [6].

S2. Challenges faced by ODE models

Power-law based systems of differential equations have numerous practically important advantages, but they also face the same intrinsic challenges as all other types of ODE models. Some of these are mentioned in this section. Generically, modeling issues can arise in a variety of biologically relevant phenomena, including generic discreteness, delays, and different manifestations of stochasticity, some of which are discussed in the Main Text and the following.

1. Generic Discreteness

Many biomedical phenomena are genuinely discrete. A simple example is the growth of a cell population, which is driven by cell division. The typical model in ODE format is exponential or logistic growth, and uncounted analyses have used these and other, similar growth "laws." Yet, these formulations predict non-integer cell numbers, which are obviously not realistic. In many instances, this type of systemic approximation error is tolerated, due to the convenience of ODE models, but it may be a problem for small numbers of cells. As a somewhat more complicated example, we analyzed the dynamics of red blood cell populations in macaques suffering from malaria (see Main Text and [36; 37]), which is genuinely discrete.

Another pertinent example is the biochemical conversion of a pool of substrate metabolites into a pool of product. This conversion in reality consists of very many distinct enzymatic steps of catalyzing one substrate molecule at a time. It is not easy to describe the time sequence of these individual steps in detail [38; 39; 40], and the overall process is therefore almost always collectively described with continuous rate functions or ODEs [41; 42], which are comparatively simple, approximately correct if sufficiently many reactions take place, and enjoy rich computational support.

More generally, the aspect of "instantaneous change," mandated by the mathematical concept of ODEs, also poses a practical, yet fundamental issue for biological modeling, namely, that it is

seldom possible to measure instantaneous changes experimentally. Measurements are naturally made on a discrete time scale, but they substitute as approximations for what is mathematically needed to formulate an ODE.

2. Delays

ODEs, by their definition, describe immediate responses to changes in input. As a pertinent, generic example, consider a simple conversion of X_1 into X_2, which occurs with a delay of $\tau = 2$ time units (Figure S1A). It may be represented with the delay differential equations (DDEs)

$$\dot{X}_1 = 1 - X_1^{0.5}(t-\tau) ,$$
$$\dot{X}_2 = X_1^{0.5}(t-\tau) - X_2 . \qquad \text{(S3)}$$

If this delayed 2-variable system starts at its steady state and X_1 is increased at a later time point, X_2 does not respond immediately, but only after τ time units (Figure S1D and E). Often, the time delay is simply ignored (Figure S1B, E) which, however, incurs a noticeably different response. In particular, both Y_1 and Y_2 start responding immediately. To mimic the delay with ODEs, one might incorporate into the model true or artificial intermediates, which slow down the overall reaction (in Figure S1C with four intermediates). While the steady state is unaffected by this simplification (not shown), the dynamics of the initial variable Z_1 and the final variable Z_6 are noticeably different from the delayed system, although this representation reflects the delayed system better than the system ignoring the delay (Figure S1E). A distinct alternative to using DDEs is a Padé approximation with a system of ODEs that is augmented with a fairly large number of auxiliary variables [43; 44]. This solution reflects the delay much better than the inclusion of intermediates but is still not 100% accurate [43].

This genuine feature of delayed ODEs is clearly an approximation of the true biological process, in which some delay occurs before the input causes a change in a variable downstream. The systemic approximation error is often tolerated because actual data are usually subject to uncertainties caused by experimental error and other limitations. However, the approximation is fundamentally at odds with reality.

In the Main Text and elsewhere [36; 37] we discussed the phenomenon of malarial anemia that inextricably involves delays due to the long lifespan of red blood cells, which ultimately rendered ODEs essentially impractical. A second pertinent example is a physiologically-based pharmacokinetic model, which describes the distribution of a drug throughout the body [45]. In such a model, every variable representing the concentration of the drug in a given organ shows an immediate, although typically small, change as soon as the drug enters the body, for instance, through an injection. Obviously, this result is unrealistic, but the discrepancies are accepted due to the convenience of ODE models.

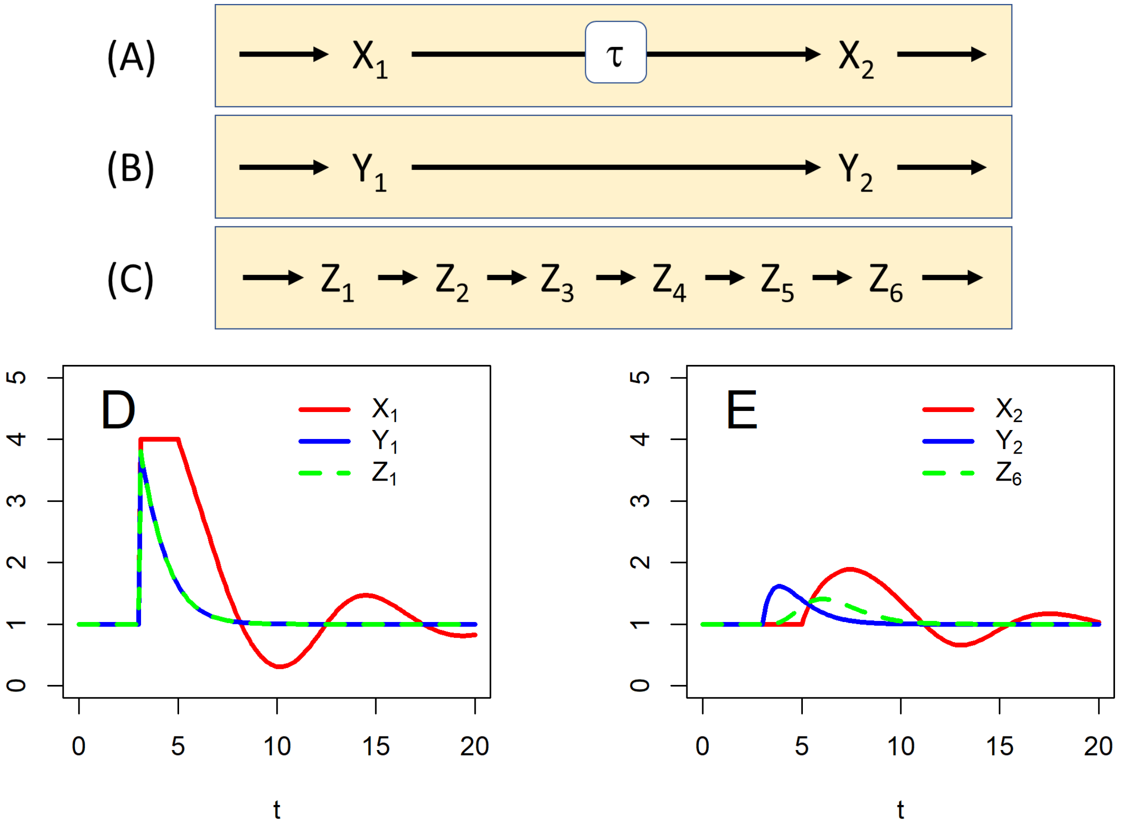

Figure S1: (A) The conversion of X_1 into X_2 with time delay τ is often modeled by simply ignoring the delay (B) or by inserting true or artificial intermediates (C). However, the dynamic responses of the three system representations, for instance, to a step-increase in the first variable at t = 3 are different. (D) Whereas X_1 of the delayed system remains constant for τ = 2 time units before starting to decrease (here modeled directly with the DDE system in Eq. (S3)), Y_1 and Z_1 start decreasing immediately. (E) The responses in the output variables of the three systems differ even more substantially. It takes τ = 2 time units before X_2 reacts at all, whereas Y_2 and Z_6 start to respond immediately. Furthermore, Y_2 and Z_6 display trajectories that are quite different from that of X_2, even missing the damped oscillations of the delayed system.

Numerous other examples incur issues caused by delays. For instance, changes in gene expression following a signal are instantaneous in ODE systems, even though they happen in reality after delays that can be substantial (see Case Study in Main Text and [46]). Another famous example is the Lotka-Volterra model describing the dynamics of interacting populations [47; 48]. While this model structure has been very successful at a coarse level, for instance for capturing the interactions between a single predator and a single prey, killing a prey leads in the model to an immediate increase in the predator population, which is clearly not the case in the actual biological system.

Discrete approaches permit both immediate and delayed changes, where “immediate” now means a change observed after a defined time step. In contrast to ODEs, these approaches by their nature do not provide information of the states of variables within these time steps.

3. Stochastic Events

By and large all biological systems are exposed to fluctuations in their environments, whose nature can often not truly be determined and which therefore might appear to be stochastic events. Generically, stochasticity emerges in two biomedically pertinent situations. First, the system may be exposed to external events that are present or absent in time intervals of random length, and to which the system responds in different ways, depending on the current input. The second situation pertains particularly to systems with relatively small numbers per modeled species, where the use of rates, for instance, for transport, metabolic conversions, or other processes, is somewhat problematic. In the case of metabolism, the catalysis of a substrate occurs in the form of a sequence of stochastic events. Capturing this non-continuous catalytic process adequately requires stochastic differential equation systems, which are ultimately based on the chemical master equation and computationally very costly [38; 40] or could be addressed with the methods of Itô calculus [49]. Even with extensions or approximations, such as the so-called tau-leaping method [39], these approaches are computationally so expensive that they become practically infeasible for large reaction systems.

The Main Text outlines examples in these categories.

4. Piecewise Dynamics

In some instances, the dynamics of a system is represented with different formulae for different sections of the time domain. If the breakpoints between these domains are known, the implementation in the format of an ODE system is straightforward. It is even possible to optimize the location of breakpoints for the minimization of the overall approximation error [50; 51]. However, if the breakpoints depend on the values of one or more of the dependent variables, the situation is more complicated. The need for this case was illustrated in [52], where the format of a rate function is thought to depend on the value of one of the variables. In this article, only the steady states for the different domains are of interest, but to capture the full dynamics of such a system, if-statements have to be embedded into the solution code for every time step, which is inconvenient and often requires knowledge of the inner workings of numerical solvers.

The Main Text provides a detailed example (*cf.* Fig. 7).

S3. Similarities between Discrete Models and ODEs

The generic format of time-discrete recursive systems is:

$$\mathbf{X}_q = F(\mathbf{X}_{q-1}, \mathbf{U}_{q-1}). \qquad \text{(S4)}$$

Here $\mathbf{X}_q$ is the state vector of the system at iteration q and $\mathbf{U}_q$ is a time-dependent vector of external influences [7; 53; 54]. Many variations of this format are possible. For instance, **X** may depend on more than one earlier state of the system. In the formulation of Eq. (S4), the iterations are not necessarily coupled to specific time steps, which are usually, although not always, regularly spaced. To enforce this coupling, a fixed time step ϑ may be defined and the equation then reads

$$\mathbf{X}_{q\cdot\vartheta} = F(\mathbf{X}_{(q-1)\cdot\vartheta}, \mathbf{U}_{(q-1)\cdot\theta}). \qquad \text{(S5)}$$

To use the advantages of the power-law approximation, we use the same philosophy as in BST by describing the change in the state of a system with a sum of products of power-law functions. Thus, to convert the typical Generalized Mass Action (GMA) system for n dependent variables, as given in Eq. (S1), into a specific type of discrete BST system in the analogous format, we define the change from one time point to the next as

$$X_{i,q\cdot\vartheta} = X_{i,(q-1)\cdot\vartheta} + \vartheta\left[\sum_{k=1}^{T_i} \pm\gamma_{ik} \prod_{j=1}^{n} X_{j,(q-1)\cdot\vartheta}^{f_{ikj}}\right] \qquad \text{(S6)}$$

for each dependent variable X_i and q = 1, 2, … , where the term in brackets is in GMA format. Note that the kinetic orders f_{ikj} are the same as in BST, but the rate constants in dBST are values scaled by ϑ and therefore depend on the size of the time step between iterations q-1 and q. Indeed, the biological meaning of rate constants in BST and dBST is slightly different.

The formulation in (S6) makes several aspects of dBST evident:

1. As in BST, the choice of suitable functions for the right-hand sides of the ODEs or the recursive equations (*cf.* (S5)) is substituted by the power-law format, which corresponds to a Taylor linearization in logarithmic variables [20]. This format is *a priori* determined, if it is known which variables affect the change in X_i directly, while the numerical values of the parameters are specific for the approximated function.

2. If $X_{i,(q-1)\cdot\vartheta}$ is moved to the left-hand side, both sides are divided by ϑ, and ϑ decreases in the limit to 0, Eq. (S6) converges toward a traditional BST equation, because

$$\lim_{\vartheta\to 0} \frac{X_{i,q\cdot\vartheta} - X_{i,(q-1)\cdot\vartheta}}{\vartheta} = \dot{X}_i = \sum_{k=1}^{T_i} \pm\gamma_{ik} \prod_{j=1}^{n} X_{j,(q-1)\cdot\vartheta}^{f_{ikj}} \qquad \text{(S7)}$$

In fact, dBST corresponds to a Euler solution of the ODEs in BST format.

3. The steady state of a recursive system is defined by $X_{i,stst} = X_{i,q\cdot\vartheta} = X_{i,(q-1)\cdot\vartheta}$, for i = 1, …, n and $q \to \infty$. Upon equating $X_{i,q\cdot\vartheta}$ and $X_{i,(q-1)\cdot\vartheta}$ in Eq. (S6) and division by ϑ, this steady-state is characterized by the same equations as a GMA system in BST. In particular, if one chooses the S-system format within BST (Eq. S2), instead of the GMA terms in Eq. (S6), and thus defines

$$X_{i,q\cdot\vartheta} = X_{i,(q-1)\cdot\vartheta} + \vartheta\left[\alpha_i \prod_{j=1}^{n} X_{j,(q-1)\cdot\vartheta}^{g_{ij}} - \beta_i \prod_{j=1}^{n} X_{j,(q-1)\cdot\vartheta}^{h_{ij}}\right] \quad \text{(S8)}$$

the steady-state for each variable X_i is directly given by

$$\alpha_i \prod_{j=1}^{n} X_{j,stst}^{g_{ij}} = \beta_i \prod_{j=1}^{n} X_{j,stst}^{h_{ij}} \quad \text{(S9)}$$

which is exactly the same as in BST and leads to a system of linear algebraic equations, if one defines logarithmic variables $y_i = \ln(X_i)$ for *i* = 1, …, *n* [8].

4. If the steady state can be computed algebraically, which is in general the case for regular S-systems with $\alpha_i \neq 0,\ \beta_i \neq 0$, the stability of this state is determined by whether $\alpha_i \prod_{j=1}^{n} X_{j,q}^{g_{ij}} - \beta_i \prod_{j=1}^{n} X_{j,q}^{h_{ij}}$ converges to 0 for $q \to \infty$ upon small perturbations, which is analogous to the criterion in BST [12].

5. It is noted that the meaning of each multiplicative parameter is not truly the same in BST and dBST, because the former represents instantaneous rates, whereas the latter describes stepwise changes.

References

[1] G. Nicolis, Introduction to Nonlieanr Science, Cambridge University Press, Cambridge, 1995.

[2] M. Cascante, A. Sorribas, R. Franco, and E.I. Canela, Biochemical systems theory: increasing predictive power by using second-order derivatives measurements. Journal of theoretical biology 149 (1991) 521-35.

[3] M.A. Savageau, Biochemical Systems Analysis: A Study of Function and Design in Molecular Biology, Addison-Wesley Pub. Co. Advanced Book Program (reprinted 2009), Reading, Mass, 1976.

[4] N.V. Torres, and E.O. Voit, Pathway Analysis and Optimization in Metabolic Engineering, Cambridge University Press, Cambridge, U.K., 2002.

[5] E.O. Voit, Modelling metabolic networks using power-laws and S-systems. Essays in biochemistry 45 (2008) 29-40.

[6] E.O. Voit, Biochemical Systems Theory: A review. Int. Scholarly Res. Network (ISRN – Biomathematics) Article 897658 (2013) 1-53.

[7] E.O. Voit, A First Course in Systems Biology (2nd Ed.), Garland Science, New York, NY, 2017.

[8] M.A. Savageau, Biochemical systems analysis. II. The steady-state solutions for an n-pool system using a power-law approximation. Journal of theoretical biology 25 (1969) 370-9.

[9] M.A. Savageau, and E.O. Voit, Recasting nonlinear differential equations as S-systems: A canonical nonlinear form. Mathem Biosci 87 (1987) 83-115.

[10] E.O. Voit, and M.A. Savageau, Equivalence between S-systems and Volterra-systems. Mathem. Biosci. 78 (1986) 47-55.

[11] I. Dattner, H. Ship, and E.O. Voit, Separable nonlinear least-square parameter estimation for complex dynamic systems. Complexity (2020) Article ID 6403641.

[12] M.A. Savageau, The behavior of intact biochemical control systems. Curr. Topics Cell. Regulation 6 (1972) 63-129.

[13] R.M.E. May, Theoretical Ecology, Principles and Applications, W.B. Saunders Co., Philadelphia, 1976.
[14] P.J. Wangersky, Lotka-Volterra Population Models. Ann. Rev. Ecol. Syst. 9 (1978) 189-218.
[15] E.O. Voit, and I.-C. Chou, Parameter estimation in canonical biological systems models. Int. J. Syst. Synth. Biol. 1 (2010) 1-19.
[16] C.M. Guldberg, and P. Waage, Studier i affiniteten. Forhandlinger i Videnskabs-Selskabet i Christiania 35 (1864).
[17] C.M. Guldberg, and P. Waage, Études sur les affinites chimiques, Brøgger & Christie, Christiania, 1867.
[18] C.M. Guldberg, and P. Waage, Über die chemische Affinität. Erdmann's Journal für practische Cehmie 127 (1879) 69-114.
[19] E.O. Voit, H.A. Martens, and S.W. Omholt, 150 years of the mass action law. PLoS Comp. Biol. 11 (2015).
[20] M.A. Savageau, Biochemical systems analysis. I. Some mathematical properties of the rate law for the component enzymatic reactions. Journal of theoretical biology 25 (1969) 365-9.
[21] M.A. Savageau, Biochemical systems analysis. 3. Dynamic solutions using a power-law approximation. Journal of theoretical biology 26 (1970) 215-26.
[22] D.H. Irvine, and M.A. Savageau, Network regulation of the immune response: alternative control points for suppressor modulation of effector lymphocytes. J Immunol 134 (1985) 2100-2116.
[23] J. Garcia, and N. Torres, Mathematical modelling and assessment of the pH homeostasis mechanisms in *Aspergillus niger* while in citric acid producing conditions. Journal of theoretical biology 282 (2011) 23-35.
[24] H. Sasidharakurup, N. Melethadathil, B. Nair, and S. Diwakar, A Systems Model of Parkinson's Disease Using Biochemical Systems Theory. OMICS 21 (2017) 454-464.
[25] M. Faraji, L.L. Fonseca, L. Escamilla-Trevino, J. Barros-Rios, N.L. Engle, Z.K. Yang, T.J. Tschaplinski, R.A. Dixon, and E.O. Voit, A dynamic model of lignin biosynthesis in Brachypodium distachyon. Biotechnol Biofuels 11 (2018) 253.
[26] T. Johnson, Estimation and simulation of S-systems. Math. Computer Modeling 11 (1988) 134-139.
[27] D.H. Irvine, and M.A. Savageau, Efficient solution of nonlinear ordinary differential equations expressed in S-system canonical form. SIAM J. Numer. Anal. 27 (1990) 704-735.
[28] P.F. Rust, and E.O. Voit, Statistical densities, cumulatives, quantiles, and power obtained by S-system differential equations. J Americ Stat Assoc (JASA) 85 (1990) 572-578.
[29] E.O. Voit, The S-distribution. A tool for approximation and classification of univariate, unimodal probability distributions. Biometrical J 34 (1992) 855-878.
[30] J.K. Horner, and M.A. Wolinsky, A power-law sensitivity analysis of the hydrogen-producing metabolic pathway in *Chlamydomonas reinhardtii*. Int. J. Hydrogen Energy 27 (2002) 1251-1255.
[31] A. Ervadi-Radhakrishnan, and E.O. Voit, Controllability of non-linear biochemical systems. Mathematical biosciences 196 (2005) 99-123.
[32] T. Zhang, Dynamics modeling of hydrogen production by sulfur-deprived Chlamydomonas reinhardtii culture in tubular photobioreactor. Int. J. Hydrogen Energy 36 (2011) 12177-12185.
[33] N. Adouani, L. Limousy, T. Lendormi, E.O. Voit, and O. Sire, Simulation of the denitrification process of waste water with a biochemical systems model: A non-conventional approach. Int. J. Chem. Reactor Eng. 12 (2014) 683-693.
[34] N.T. Fortun, Analysis of the earth's carbon cycle models using biochemical systems theory and chemical reaction network theory, De La Salle University, Manila, Philippines, 2018.
[35] M. Iwata, A. Miyawaki-Kuwakado, E. Yoshida, S. Komori, and F. Shiraishi, Evaluation of an S-system root-finding method for estimating parameters in a metabolic reaction model. Mathematical biosciences 301 (2018) 21-31.

[36] L. Fonseca, A. H.A., A. Moreno, J.W. Barnwell, M.R. Galinski, and E.O. Voit, Quantifying the removal of red blood cells in Macaca mulatta during a Plasmodium coatneyi infection. Malaria J. (2016) 410.
[37] L. Fonseca, and E.O. Voit, Comparison of mathematical frameworks for modeling erythropoiesis in the context of malaria infection. Mathematical biosciences 270 (2015) 224-236.
[38] D.T. Gillespie, A general method for numerically simulating the stochastic time evolution of coupled chemical reactions. J. Comp. Physics 22 (1976) 403–434.
[39] D.T. Gillespie, Stochastic simulation of chemical kinetics. Ann. Rev. Phys. Chem. 58 (2007) 35-55.
[40] O. Wolkenhauer, M. Ullah, W. Kolch, and K.-H. Cho, Modeling and simulation of intracellular dynamics: Choosing an appropriate framework. IEEE Trans. NanoScience 1 (2004) 200-207.
[41] L.A. Segel, Biological Kinetics, Cambridge University Press, Cambridge, UK, 1992.
[42] H.M. Sauro, Enzyme Kinetics for Systems Biology, Ambrosius Publishing, 2012.
[43] W.T. Mocek, R. Rudnicki, and E.O. Voit, Approximation of delays in biochemical systems. Mathematical biosciences 198 (2005) 190-216.
[44] M. Vajta, Some remarks on Padé-approximation. 3rd TEMPUS-INTCOM Symposium September 9-14, Veszprém, Hungary (2000).
[45] J.E. Sager, J. Yu, I. Ragueneau-Majlessi, and N. Isoherranen, Physiologically Based Pharmacokinetic (PBPK) Modeling and Simulation Approaches: A Systematic Review of Published Models, Applications, and Model Verification. Drug Metab Dispos 43 (2015) 1823-37.
[46] T. Gedeon, and P. Bokes, Delayed protein synthesis reduces the correlation between mRNA and protein fluctuations. Biophys J 103 (2012) 377-385.
[47] A.J. Lotka, Contribution to the Theory of Periodic Reaction. J. Phys. Chem. 14 (1910) 271–274.
[48] V. Volterra, Variazioni e fluttuazioni del numero d'individui in specie animali conviventi. Mem. R. Accad. dei Lincei. 2 (1926) 31-113.
[49] C. Rogers, and D. Williams, Diffusions, Markov processes and martingales - Volume 2: Itô calculus, Cambridge University Press, Cambridge, 2000.
[50] G. Ferrari-Trecate, and M. Muselli, A new learning method for piecewise linear regression. in: J.R. Dorronsoro, (Ed.), Lecture Notes In Computer Science: Proceedings of the International Conference on Artificial Neural Networks, Springer Verlag, Berlin, 2002, pp. 44 - 449.
[51] A. Machina, A. Ponosov, and E.O. Voit, Automated piecewise power-law modeling of biological systems. J. Biotechnology invited (2009).
[52] M.A. Savageau, Design principles for elementary gene circuits: Elements, methods, and examples. Chaos 11 (2001) 142-159.
[53] L. Edelstein-Keshet, Mathematical Models in Biology, Society of Industrial and Applied Mathematics, Philadelphia, 2005.
[54] R. Robeva, (Ed.), Algebraic and Discrete Mathematical Methods for Modern Biology, Academic Press, Amsterdam, 2015.
